# Comparing the Protective Effects of Curcumin and Ursodeoxycholic Acid after Ethanol-Induced Hepatotoxicity in Rat Liver

**DOI:** 10.4314/ejhs.v31i3.25

**Published:** 2021-05

**Authors:** Azam Letafat Farashbandi, Mehrdad Shariati, Mokhtar Mokhtari

**Affiliations:** 1 Department of Biology, Kazerun Branch, Islamic Azad University, Kazerun, Iran

**Keywords:** Curcumin, Ursodeoxycholic acid, Ethanol, Hepatotoxicity, Rat

## Abstract

**Background:**

Alcohol consumption can cause hepatitis and long-term cirrhosis of the liver. The aim of this study was to evaluate the protective effects of curcumin (CUR) and ursodeoxycholic acid (UDCA) alone and together in the prevention and treatment of liver damage caused by overuse of ethanol.

**Methods:**

Adult Wistar rats were divided into 8 groups of 5, including the control group and various combinations of ethanol, CUR and UDCA groups. Twenty-eight days after the oral treatment, serum levels of aspartate transaminase (AST), alanine transaminase (ALT), alkaline phosphatase (ALP), gamma-glutamyl transferase (GGT) and Arginase I (ArgI) as well as serum levels of Albumin (Alb), total protein (TP) and Blood Urea Nitrogen (BUN) were measured, and liver tissue was evaluated histopathologically.

**Results:**

The solo administration of CUR, UDCA and CUR+UDCA had no effect on the blood parameters and liver tissue compared to the control group (p>0.05). The solo administration of CUR and UDCA in ethanol-treated rats significantly reduced ALT, AST, ALP, GGT, ArgI and BUN levels (p<0.05), while the solo administration increased Alb and TP levels compared to the ethanol group (p<0.05). In these groups, a significant decrease in cell necrosis and local inflammation of hepatocytes was observed, and the liver damage was mild. However, co-administration of ethanol, CUR and UDCA made significantly greater decrease in ALT, AST, ALP, GGT, ArgI and BUN levels (p>0.05), while the co-administration greatly increased Alb and TP levels compared to the ethanol group (p<0.05). Histopathologically, a decrease in structural changes in liver tissue and inflammation was observed, resulting in the improvement of liver tissue.

**Conclusion:**

The solo administration of CUR and UDCA could reduce ethanol-induced liver damage in rats and improve liver's serum and blood parameters. However, the coadministration of CUR and UDCA has a greater efficacy.

## Introduction

Acute liver failure is caused by a number of factors, including viral hepatitis, toxic damages from toxins and drugs, and ischemia (1). The liver is the body's first defense barrier, constantly exposed to various types of internal and external toxins in high concentrations. Oxidative stress plays a key role in liver damage (2). Due to the fact that alcohol is consumed as a beverage in most countries of the world, it can cause liver damage in such a way that its excessive consumption harms both physical and mental health (3). The liver is the main site of ethanol metabolism and is also the main target organ for ethanol-induced damage (4). Excess ethanol consumption can trigger the progression of alcoholic liver disease, which ranges from fibrosis to liver Cirrhosis. Oxidation stress plays a major role in the pathogenesis of alcoholic liver damage. Ethanol consumption increases reactive oxygen species (ROS) and decreases glutathione (GSH) and superoxide dismutase (SOD) levels, thereby reducing antioxidant capacity in target tissues (5). Recent studies have shown that antioxidant therapy prevents liver damage due to ethanol consumption. Therefore, maintaining antioxidant capacity is expected to reduce liver damage due to ethanol consumption (6).

The clinical and animal studies have represented that inflammatory cytokines such as TNF-α and IL-6 is major mediators of ethanolinduced liver damage. In addition, regarding the researchers, the cathepsin B pathway mediates apoptosis via TNF-α in hepatocytes (7). It has also been reported that reducing TNF-α and IL-6 levels by inhibiting oxidative activity can reduce ethanol-induced hepatitis. The clinical and animal studies have shown that antioxidants present in food can protect the body against various types of oxidative damage caused by free radicals. In traditional medicine, turmeric (Curcuma longa) has been mentioned as a laxative, bile and protective plant of the liver. Given that the use of herbal medicines is increasing in recent years, it is logical to review and revive old medicines, and understanding the mechanism of their action can lead to the formation and acceptance of new therapies for many diseases (8). Turmeric is a widely used plant in Ginger family and is composed of a wide range of phytochemicals including curcumin, dimethoxy curcumin, bis-di-methoxy curcumin, curcuminol, curcumol tetrahydrocurcumin, turmeric, turmerons and turmeronol (9). The most important constituents of turmeric are curcuminoids, which also produce the yellow color of turmeric (10). Turmeric has different biological activities. For example, the aqueous extract of turmeric has antioxidant and anti-inflammatory activity. It also heals corneal ulcers and regulates cytochrome P450 (CYP) activity. Recently, it has been shown that turmeric hot water extract regulates the adhesion characteristic of endothelial cells by inhibiting TNF-α and preventing the transmission of the NF-κB signal (11). Curcumin (CUR) is a turmeric compound that has both antioxidant and anti-inflammatory activities (12). Curcumin is a polyphenolic compound and an active ingredient derived from the rhizome of the turmeric plant that exerts effective antioxidant, anti-inflammatory and antimicrobial activity and it has been shown that daily consumption of curcumin may have a protective effect on the liver against oxidative stress associated with alcohol consumption (13,14).

Ursodoxycholic acid (UDCA) is a natural bile acid that, while weakening the production and secretion of cholesterol, inhibits intestinal absorption of cholesterol and plays an important role in altering the rate of apoptosis in cells. Several mechanisms have been proposed to reduce apoptosis by bile acids, including decreased cytochrome-*c* release from mitochondria and reduced FAS ligand-induced apoptosis. Ursodoxycholic acid seems to reduce bile acid detergent and reduce hepatotoxicity in people with chronic hepatic cholestatic disease. In chronic liver disease in which the concentration of bile acids increases, the consumption of this drug protects the liver cell against the toxic effects of bile acids (15). As far as CUR has antioxidant and anti-inflammatory activity and UDCA has anti-apoptotic and cholesterol inhibitory activity, this study was designed to investigate the effect of CUR and UDCA on ethanol-induced liver damage in rats.

## Methods

**Animals:** In this study, 64 adult male Wistar rats with an average weight of 290± 10 g and 9 weeks old were prepared from the animal house of Islamic Azad University of Kazerun and kept. Before the experiment and in order to adapt to the new conditions, the animals were kept together for 7 days and fed with a basic diet. The animals were kept in a controlled environment with a temperature of 23 ±1 C, relative humidity of 55±5% and a cycle of 12 hours of darkness and 12 hours of light. During this study, the animals had adequate access to food and water based on the basic diet of (AIN)-93G American Institution. Based on the guidelines for working with laboratory animals, the protocol of this study was prepared and approved by the Ethics Committee of the Azad University, Kazerun Branch (Ethical code number: No: IR.Kiau 15570).

**Study protocol**: The doses of ethanol, CUR and UDCA were selected based on previous studies (4,7,16). Animals were randomly divided into 8 groups of 5 including control, Ethanol30 (as positive control group), CUR100 (as negative control group 1), UDCA20 (as negative control group 2), UDCA20+CUR100 (as negative control group 3), Ethanol30+CUR100 (as experimental group 1), Ethanol30+UDCA20 (as experimental group 2) and Ethanol30+CUR100+UDCA20 (as experimental group 3) as follows:

1- Control group: did not receive any drug treatment for 28 days.

2- Ethanol30 group: received 2 ml of ethanol 30% orally at 8 a.m.

3- CUR100 group: received 100 mg/kg of CUR orally at 4 p.m.

4- UDCA20 group: received 20 mg/kg UDCA orally at 4 p.m.

5- CUR100+UDCA20 group: received 100 mg/kg CUR at 4 p.m and 20 mg/kg UDCA orally at 8 p.m.

6- Ethanol30+CUR100 group: received 2 ml of ethanol 30% at 8 a.m. and 100 mg/kg of CUR orally at 4 p.m.

7- Ethanol30+UDCA20 group: received 2 ml of ethanol 30% at 8 a.m. and 20 mg/kg UDCA orally at 4 p.m.

8- Ethanol30+CUR100+UDCA20 group: received 2 ml ethanol 30% at 8 a.m, 100 mg/kg CUR at 4 p.m. and 20 mg/kg UDCA orally at 8 p.m.

At the end of the study, all animals were first anesthetized with diethyl ether (Merck, Germany) and then blood samples were taken directly from the heart using a 5 cc syringe. Blood samples were placed in an incubator (Memmert UNB 400, Germany) for 30 minutes at 37 ° C. After agglutination, the tubes were placed in a centrifuge (MSE, England) for 10 minutes at 2500 rpm to separate the serum. Serum levels of AST, ALT, ALP, GGT, ArgI, Alb, TP and BUN were measured by RA-1000 model auto-analyzer (Technicon, USA) according to the manufacturer's instructions (Pars Azmoun Co, Iran). Serum ALT and AST levels were measured by International Federation of Clinical Chemistry (IFCC) method without adding Pyridoxal-50phosphate. Serum ALP level by Deutsche Gesellschaft Fur Klinische Chemie (DGKC) method, serum GGT level by enzymatic method according to Szasz method and serum ArgI level were measured by Urease- glutamate dehydrogenase (Urease-GLDH) method. Also, serum TP level was measured by photometric method based on Biuret method; serum Alb level was measured by Bromocresol-Green (BCG) method and serum BUN level was measured by 2,4-Dichloroaniline (DCA) method. After blood sampling, by opening the abdominal area, the liver tissue of all animals was removed and placed in 10% formalin buffer solution for fixation. After tissue passage, the samples were molded in paraffin, and serial sections were prepared for histopathological study using a microtome device.

**Preparing ethanol**: Using Pasteur pipette, 30 ml of ethanol 99% (Ghadir Ethanol Industries Co, Iran) was mixed with 70 ml of distilled water to obtain ethanol 30% solution.

**Preparing curcumin**: The turmeric plant was dried at 70°C for 24 hours and then was grounded using a mill. The resulting powder was sieved with mesh sizes of 40–80 to obtain a powder with a uniform distribution of 0.18–0.24 mm. The resulting powder was stored in refrigerator to prevent moisture absorption. Then, one gram of dried turmeric powder was mixed with 30 grams of carbamate ion liquid (Adonis Gol Daroo Co, Iran) and stirred in an incubator shaker at 15–45°C for 1–8 hours. The mixture was centrifuged (5 min, 1500 rpm), and the supernatant was filtered using a milli-pour system. The filtered liquid was distilled at 40°C using a vacuum rotary evaporator, and the carbonate ionic liquid was recovered. The Oleoresin (Adonis Gol Daroo Company, Iran) obtained from the extraction process was purified by column chromatography to obtain high purity Curcumin.

**Ursodeoxycholic acid**: Ursodoxycholic acid is a natural bile acid found in small amounts in the bile that weakens the production and secretion of cholesterol by the liver and inhibits intestinal absorption of cholesterol. This drug was prepared in the form of tablets from Sigma Company, USA.

**Statistical analysis**: In this study, the obtained data were analyzed using SPSS software version 20 (SPSS Inc, Chicago, IL, USA). First, the normal distribution of data was confirmed using Kolmogorov-Smirnov test, and then, the differences between means were analyzed at the level of P<0.05 using one-way analysis of variance, followed by the least significant difference (LSD) *post-hoc* test for multiple comparison. Using GraphPad Prism software version 6 (GraphPad Prism, Inc., San Diego, CA, USA), the results were expressed as mean ± standard deviation in the graphs.

## Results

**Figure 1:**
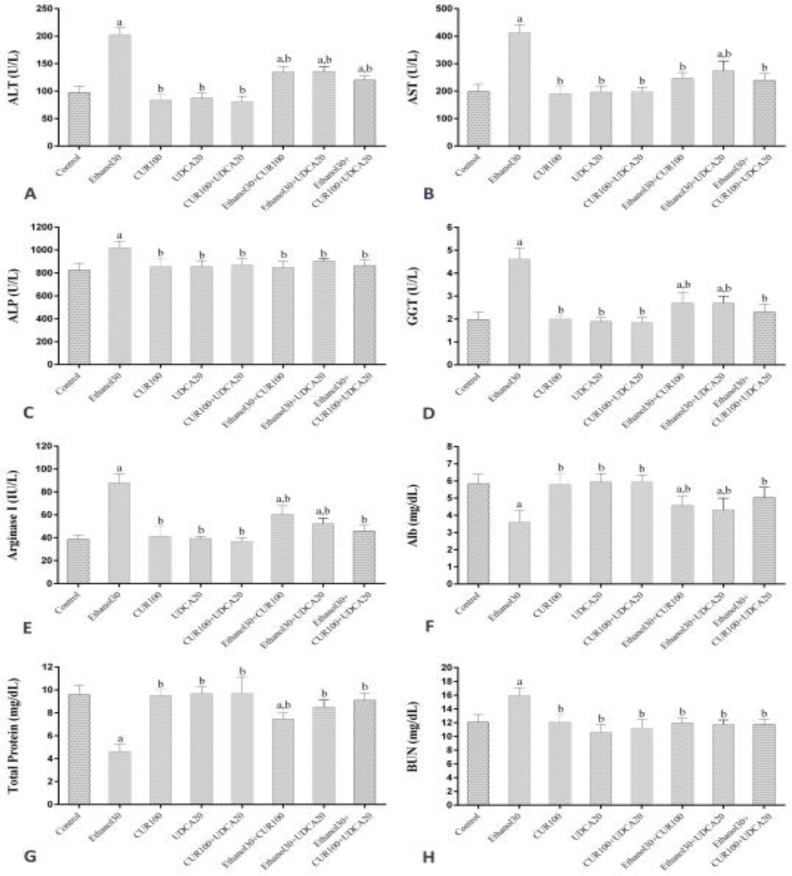
Comparison of mean and standard deviation of serum levels of ALT, AST, ALP, GGT, ArgI, Alb, TP and BUN in control groups, Ethanol30, CUR100, UDCA20, UDCA20+CUR100, Ethanol30+CUR100, Ethanol30+UDCA20 and Ethanol30 a and b: p<0.05, as compared with Control and Ethanol30 groups, respectively

**Biochemical findings**: Figure 1 shows the results of administrating CUR, UDCA and ethanol on serum ALT, AST, ALP, GGT, ArgI, Alb, TP and BUN level in different groups. Serum ALT level (Figure 1A) in the Ethanol30 group showed a significant increase compared to the control, CUR100, UDCA20, CUR100+UDCA20, Ethanol30+CUR100, Ethanol30+UDCA20 and Ethanol30+CUR100+UDCA20 groups (P<0.05). There was no significant difference in ALT level between CUR100, UDCA20 and CUR100+UDCA20 and control groups (P>0.05), but Ethanol30+CUR100, Ethanol30+UDCA20 and Ethanol30+CUR100+UDCA20 groups had a significant increase compared to the control group (P<0.05). Serum AST levels (Figure 1B) in the Ethanol30 group showed a significant increase compared to the control, CUR100, UDCA20, UDCA20+CUR100, Ethanol30+CUR100, Ethanol30+UDCA20 and Ethanol30+CUR100+UDCA20 groups (P<0.05). There was no significant difference in AST level between CUR100, UDCA20, UDCA20+CUR100, Ethanol30+CUR100 and Ethanol30+CUR100+UDCA20 groups and control groups (P>0.05), but Ethanol30+UDCA20 group had a significant decrease compared to the control group (P<0.05).

Serum ALP level (Figure 1C) in Ethanol30 group showed a significant increase compared to the control group (P<0.05) while the other groups did not differ significantly from the control group (P<0.05). Serum GGT and ArgI levels (Figure 1D and 1E, respectively) in the Ethanol30 group showed a significant increase compared to the control group, CUR100, UDCA20, UDCA20+CUR100, Ethanol30+CUR100, Ethanol30+UDCA20 and Ethanol30+CUR100+UDCA20 (P<0.05). CUR100, UDCA20, UDCA20+CUR100 and Ethanol30+CUR100+UDCA20 groups had no significant differences in serum levels of GGT and ArgI with the control group (P>0.05), but Ethanol30+CUR100 and Ethanol30+UDCA20 groups had a significant increase compared to the control group (P< 0.05).

Serum Alb level (Figure 1F) in the Ethanol30 group showed a significant decrease compared to the control, CUR100, UDCA20, UDCA20+CUR100, Ethanol30+CUR100, Ethanol30+UDCA20 and Ethanol30+CUR100+UDCA20 groups (P<0.05). CUR100, UDCA20, UDCA20+CUR100 and Ethanol30+CUR100+UDCA20 groups were not significantly different from the control group in Alb serum level (P>0.05), but Ethanol30+CUR100 and Ethanol30+UDCA20 groups had a significant decrease compared to the control group (P<0.05). Serum TP level (Figure 1G) in the Ethanol30 group showed a significant decrease compared to the control groups, CUR100, UDCA20, UDCA20+CUR100, Ethanol30+CUR100, Ethanol30+UDCA20 and Ethanol30+CUR100+UDCA20 (P<0.05). CUR100, UDCA20, UDCA20+CUR100, Ethanol30+UDCA20 and Ethanol30+CUR100+UDCA20 groups did not differ significantly in serum TP level from the control group (P>0.05), but Ethanol30+CUR100 group had a significant decrease compared to the control group (P<0.05). Serum BUN level (Figure 1H) in the Ethanol30 group showed a significant increase compared to the control group (P<0.05) while the other groups did not differ significantly from the control group (P>0.05).

**Figure 2:**
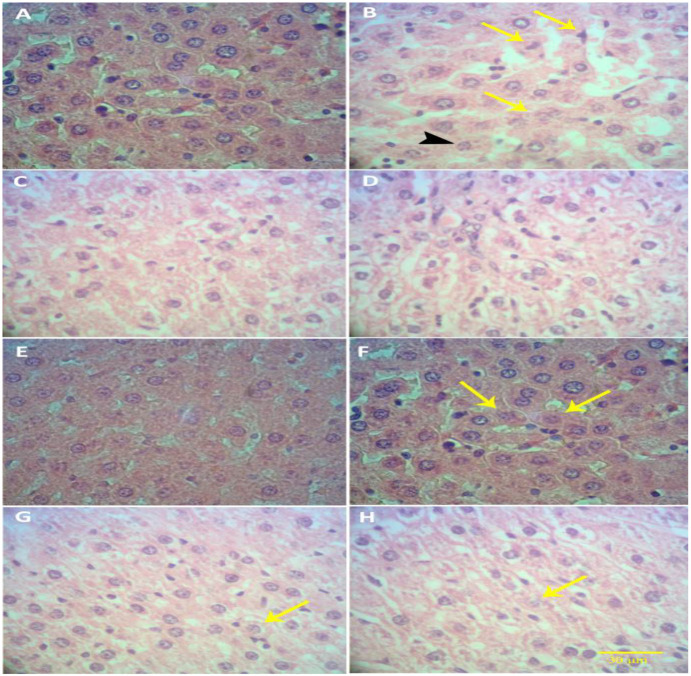
**Optical photomicrograph of liver tissue-** A: In the control group, the normal structure of liver tissue is observed. B: In the Ethanol30 group, necrosis (yellow arrows), karyorrhexis (black arrowhead), karyolysis and eosinophilia of the cytoplasm are observed. Liver tissue is destroyed. C, D and E: In groups CUR100, UDCA20 and CUR100+UDCA20, liver tissue and hepatocytes are normal. F and G: In the groups of Ethanol30+CUR100 and Ethanol30+UDCA20, cell necrosis (yellow arrows) and hepatocyte hyperemia are reduced and liver tissue destruction is mild. H: In the Ethanol30+CUR100+UDCA20 group, a very mild necrosis (yellow arrow) is observed and structural changes in liver tissue are very minor. Liver tissue is improved. (E&H staining, 40X; bar: 30 µm)

**Histopathological findings**: Figure 2 shows the histopathological findings in different groups. There were no evidence of hepatocyte and central venous damage in the control, CUR100, UDCA20 and UDCA20+CUR100 groups (Figures 2A, 2C, 2D and 2E, respectively). The hepatocytes were placed together regularly and without any damage, and the liver tissue was completely normal.

In the Ethanol30 group (Figure 2B), compared to the control group, many structural changes were observed in liver tissue. Accumulation of fat droplets was observed in Ito cells (a type of Sinusoid cells), hyperemia, necrosis, apoptosis, vacuolar resorption, and congestion in liver tissue. Also pycnosis, karyorrhexis, lysis (karyolysis) of cell nucleus and eosinophilia of the cytoplasm were observed.

In Ethanol30+CUR100 and Ethanol30+UDCA20 groups (Figure 2F and 2G, respectively), a significant decrease in cell necrosis and local inflammation of hepatocytes was observed compared to Ethanol30 group, such that hepatocyte hyperemia was significantly reduced in these groups. In both groups, the liver damage was mild. In Ethanol30+CUR100+UDCA20 group (Figure 2H), the histopathological changes were so small such that mild necrosis was observed.

## Discussion

The aim of this study was to investigate the effect of CUR and UDCA in solo and combinational forms on the level of blood parameters (ALT, AST, ALP, GGT, ArgI, Alb, TP and BUN) and changes in liver tissue in ethanol-treated rats. The results of this study showed that the solo administration of CUR (100 mg/kg) and solo administration of UDCA (20 mg/kg) in ethanol (30%)-treated rats improved the level of all blood parameters and liver tissue changes compared to ethanol30 group, but significant differences were also observed compared to the control group. However, according to the results of this study, coadministration of CUR and UDCA in ethanoltreated rats did not show any significant differences in blood parameters and liver tissue changes compared to the control rats, except for a significant increase in the ALT levels.

One of the most important liver diseases that are developing in most countries is alcoholic or ethanolic liver disease, which can be associated with the risk of death or disability. Once absorbed by the body, ethanol is extensively metabolized in the liver, and its metabolites can cause liver damage. The most important sign of liver damage and hepatotoxicity is the leakage of liver enzymes into the plasma (17). Although ethanol can cause liver damage through several pathways, the role of oxidative stress and free radical damage appears to be more prominent (18). Under natural conditions, there is a balance between the production and elimination of free radicals in the body, and ethanol can cause imbalance in these processes and cause oxidative stress by disrupting antioxidant systems and overproduction of free radicals. Free radicals can damage important macromolecules in the body, including proteins, fats, and DNA due to their tendency to absorb electrons (19, 20).

In the present study, ethanol intake caused hepatic necrosis leading to increased serum levels of ALT, AST, ALP, GGT, ArgI and BUN and in turn decreased the TP and Alb. Increased ALT, AST, ALP and GGT levels and decreased TP and Alb levels in this study indicate cell leakage and structural damage and the dysfunction of cell membranes in the liver due to oxidative stress, lipid peroxidation and inflammation (19–21). Arginase is an enzyme that is responsible for the hydrolysis of L-arginine to L-ornithine during the urea cycle and is expressed specifically in the liver of animals (22). In the liver, M2 macrophages in response to interleukin 4 and 13, release anti-inflammatory factors such as ArgI, interleukin 10, and the transforming growth factor-β, which are involved in wound healing, tissue regeneration, and fibrosis. According to the results of this study, ethanol administration increased ArgI levels, which is an anti-inflammatory reaction, in response to increased hepatic inflammation induced by ethanol (23). BUN is an important indicator of hepatic-renal function. Levels of malondialdehyde, SOD and catalase activity increase with alcohol consumption, which can cause hepatic-kidney damage (24). In this study, ethanol administration increased the BUN level that indicated hepatic-renal dysfunction.

The results of this study showed that CUR and UDCA provide hepatic protection against ethanol-induced hepatotoxicity. Xionget al. showed that the administration of CUR at doses of 75 and 150 mg/kg in ethanol-treated rats reduced ALT and AST levels (18). Salman et al. also found that the administration of CUR at a dose of 100 mg/kg in carbon tetrachloride (CCL4)-treated rats decreased ALT, AST, ALP and GGT levels while increased Alb levels (21), which is consistent with the results of this study. In the study by Arafa et al., CUR reduced ALT and AST levels by 35.5% and 57%, respectively, in diet-induced hypercholesterolemia rats (25). Curcumin has many biological activities such as anti-inflammatory, anti-diabetic and antioxidant activities and can improve cytotoxic and inflammatory effects in the liver. The studies show that CUR reduces hepatic transaminase activity by improving liver tissue structure. Curcumin exerts protective effects on various tissues of the body in two ways: 1- by antioxidant activity and 2-inhibition of NF-κB, which is a transcription factor of inflammatory mediators in the liver. Curcumin prevents and scavenges the formation of free radicals such as reactive species of oxygen and nitrogen (26). According to the results of this study, Akinyemi et al. showed that in cadmiumtreated rats, the administration of CUR at doses of 12.5 and 25 could have a protective effect on the renal toxicity by inhibiting Arg activity (27). Gozeneliet et al. also reported that the administration of CUR in thymoquinone-treated rats maintained Arg levels compared to the control group and proved the regeneration of rat livers subject to 70% hepatectomy (22). According to the results of Ghelaniet et al., the administration of CUR in doses of 100 and 150 mg/kg dramatically reduced the level of BUN and had a protective effect on the liver and renal tissue in adenine-treated rats (28), which is in line with the results of this study.

Ursodeoxycholicacid affects liver function through complex mechanisms. It also lowers bile cholesterol by inhibiting intestinal cholesterol absorption and secretion into the bile (29). Studies show that the protective effects of UDCA are due to its ability to protect hepatocytes against bile acids. Bile acids can cause inflammatory processes and impair mitochondrial function. However, UDCA can activate anti-apoptotic pathways while preserving cellular structures such as plasma membranes and mitochondria, and prevents from hepatocytes damage. Ursodeoxycholic acid can inhibit the formation of ROS in Kupffer cells and thus reduces the level of oxidative stress in liver cells (30–32). Consistent with the results of this study, the administration of UDCA for 4 weeks in patients with liver disorders reduces the levels of liver enzymes ALT, AST and GGT by 40, 34 and 23%, respectively, compared with placebo. Ursodeoxycholic acid appears to increase the synthesis of glutathione and ascorbic acid by increasing the N-acetylcysteine level. Therefore, UDCA has antioxidant capacity. Ursodeoxycholic acid can also reduce the phenylalanine/tyrosine/tryptophan levels by modulating the intestinal microbiome and improving liver function (33). The administration of UDCA in doses of 400, 800 and 1200 mg/kg for two weeks in healthy individuals and in all doses is associated with decreased levels of ALT, AST and miR-122 expression. Increased expression of miR-122 is associated with liver disorders (34). Salman et al reported that the administration of UDCA in CCL4-treated rats significantly reduced ALT, AST, ALP, GGT and Urea levels while increased Alb level, which is consistent with the results of this study. Ursodeoxycholic acid also increased the levels of GSH, SOD and catalase but decreased the levels of Malondialdehyde and nitric oxidein CCL4-treated rats by making a protective effect on liver tissue (21).

The results of this study show that, in the groups that have used CUR and UDCA simultaneously, there was an improvement in blood parameters and histopathological indices compared to the group receiving ethanol, and the indices were similar with the control group. Based on the biochemical and histopathological results of this study, it can be concluded that the role of CUR and UDCA is to prevent oxidative stress caused by ethanol in different sections of the liver. In living organisms, there are two antioxidant systems to counteract the destructive effects of free radicals and oxidative stress, including enzymatic antioxidant defense (SOD, GSH and catalase) and non-enzymatic ones including ascorbic acid, Alpha tocopherol, Billy Rubin, Uric acid and polyphenols (35–37). These compounds minimize the damage caused by free radical activity by preventing the production of free radicals and repairing and regenerating the damaged tissues. Therefore, it seems that due to their antioxidant activity and the inhibitory effects of UDCA in inhibiting the production and secretion of cholesterol in the liver, CUR and UDCA inhibit the destructive effect and oxidative properties and lipid peroxidation of ethanol and play a protective role for liver cells in ethanol-treated rats (21). In a study conducted by Palizgir et al, it was found that CUR inhibits the secretion of tumor necrosis factor-alpha (TNF-α) and the levels of ALT, AST, ALP, GGT enzymes and reduces it close to the normal level (37). Also, in a study conducted by Ghandadi et al, it was found that CUR inhibits interleukin-1, which is one of the causes of tissue necrosis made by macrophages. In this study, it was shown that CUR and UDCA can greatly inhibit the destructive effects of ethanol on the liver (38). Consistent with the results of this study, Gheibi et al. showed that the co-administration of CUR (200 mg/kg) and UDCA (80 mg/kg) reduced ALT and AST levels in rats with non-alcoholic fatty liver disease. The study also showed that the co-administration of CUR and UDCA improved liver tissue in rats with non-alcoholic fatty liver disease by increasing antioxidant capacity and anti-inflammatory responses (39).

In this study, ethanol administration caused severe histopathological changes in rat liver tissue by increasing cell inflammation for 28 days. However, CUR and UDCA in ethanol-treated rats had protective effects and improved liver tissue whereas the concomitant administration of CUR and UDCA has a greater efficacy. The protective and therapeutic effects of CUR and UDCA seem to be mediated by a reduction in oxidative stress and tissue inflammation. Therefore, concomitant administration of CUR and UDCA as a supplement can be considered in the diet of those exposed to ethanol liver poisoning.
